# The Unusual High Origin Radial Artery in a Black Kenyan Population: A Cadaveric Study

**DOI:** 10.4314/ejhs.v32i2.25

**Published:** 2022-03

**Authors:** Brian N Bundi, Victor Mutua, Isaac Cheruiyot, Jeremiah Munguti, Chris von Csefalvay, Khulud Mahmood Nurani, Julius Ogeng'o

**Affiliations:** 1 Department of Human Anatomy and Medical Physiology, University of Nairobi, Kenya; 2 Starschema Inc., Office of Intramural Research, Arlington, VA, USA

**Keywords:** Radial artery, high origin, arterial variation

## Abstract

**Background:**

The anatomy of the radial artery draws great interests among anatomists for its frequent involvement in variations. Equally, these variations have gained significant attention from clinicians because of the preference to use the radial artery for catheterization. The commonest of radial artery variations involve its site of origin. In published literature, data on this variations exist, but the prevalence of such variations in a Kenyan population has hitherto been unknown.

**Methods:**

Sixty-two upper limbs from 50 formalin-fixed cadavers were studied during dissection in the Department of Human Anatomy, University of Nairobi.

**Results:**

Fifty-four (87.1%) radial arteries arose within the cubital fossa, while eight (12.9%) had a high origin. Out of the eight high arteries, two (3.2%) branched off from the axillary artery, another two (3.2%) were branches of the proximal third of the brachial artery and four (6.5%) arose from the middle third of the brachial artery. The high origin radial arteries were more common on the right upper limbs (5 out of the 8 cases). Both axillary and brachial origins were seen bilaterally.

**Conclusion:**

The present study details important variations in the anatomy of the radial artery in a Kenyan population. With the radial artery being utilized during clinical, surgical and radiological interventions so frequently, an increased understanding and anticipation of such topographic variances is paramount.

## Introduction

The radial artery is the smaller-diameter vessel of the two terminal branches of the brachial artery. However, despite its caliber, it is considered the direct continuation of the brachial artery. Its course extends from the neck of the radius, which is the site of bifurcation of the brachial artery, and its branches are distributed to the musculature of the flexor compartment of the forearm. Proximally, the radial artery is under cover of the brachioradialis and further distally, it obtains more superficial location as it overlies the distal radius and the styloid process. In the forearm, besides giving direct branches to the superficial muscles of the flexor compartment, the vessel gives off a radial recurrent artery, palmar and dorsal carpal branches and a superficial palmar branch. Within the hand, it gives rise to the first metacarpal artery and the radialis indicis. The radial artery classically terminates in the hand just beyond the snuffbox by forming the deep palmar arch (1,2).

Variant anatomy in peripheral arteries is not uncommon and has been attributed to the multiplicity of factors influencing their development (3). Out of the major vessels in the upper limb, the radial artery is the one most commonly involved in topographic variations (4). Its variant anatomy generally centers on an unusual origin, an aberrant course, atypical termination, duplication, anastomoses or absence, (3,5–7). The high origin radial artery constitutes (87.5%) of all its variations (4). It arises proximal to the inter-epicondylar line of the humerus, from the brachial artery or the axillary artery coursing deep to the brachial fascia (8–11).

The overall population-level prevalence of the radial artery of high origin (referred to as brachioradial artery) is, however, quite diverse. While some studies report prevalences of 5.1% and 8.8% (12–14), others put the prevalence of this variation at 15%, almost twice as prevalent (15–17). This disparity has not been explained. The lower prevalences may downplay the significance of such a common anomaly, resulting in under-anticipation during clinical procedures, while the higher prevalences may exaggerate its relevance. Further, there is scant information about the origin of the radial artery in the Kenyan population.

The anatomy of the radial artery is of considerable importance, especially since transradial catheterization for coronary and intracardiac access has become the preferred alternative over the trans-femoral approach (12). In addition, knowledge of the radial artery's topography is important in surgeries involving the forearm. This is because iatrogenic injury of the vessel, especially during surgeries and intravenous instrumentation, has been known to be the most frequent complication of such procedures. Such injuries may be avoided by additional knowledge of observed variations. Thus, establishing the relative frequencies of radial artery variations is important for clinical, surgical and radiological purposes alike. We therefore set out to study the topography of radial artery among Kenyans.

## Materials and Methods

Our sample consisted of 50 cadavers that evidenced no obvious deformity or scars indicative of past surgery to the upper limb. We excluded limbs that already had been dissected during routine dissection in the Department's gross anatomy laboratory. A linear skin incision was extended from the deltopectoral groove down the forearm and up to the mid-palm. The superficial veins and musculature were carefully prosected to expose the entire length of the axillary, brachial, radial and ulnar arteries. The site of origin of the radial artery was then noted and recorded into data collection sheets.

## Results

**Figure 1:**
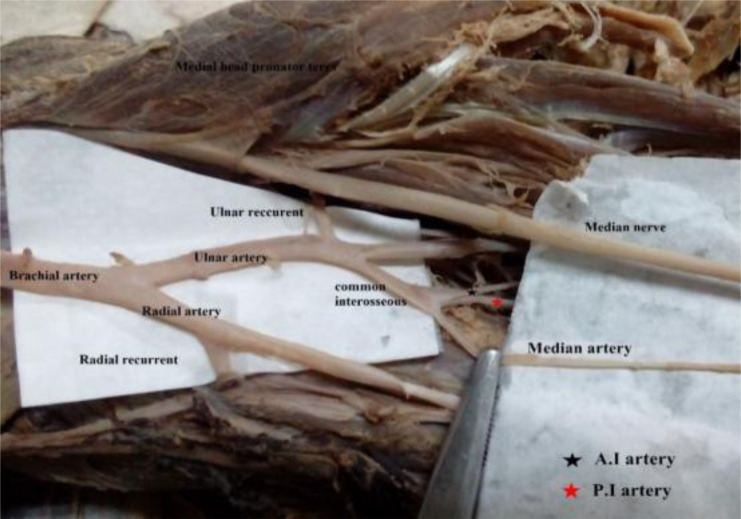
Typical arterial anatomy at the cubital fossa. Note the normal origin of the radial artery

A total of 62 limbs were studied, of which 34 (54.8%) were right limbs and 28 (45.2%) were left limbs. We observed a normal site of origin for the radial artery in 54 limbs (29 and 25 on the right and left respectively) in which the radial artery was a continuation of the brachial artery within the cubital fossa (Figure 1). A total of eight limbs showed atypical radial arteries, characterized by a high level of origin. Out of the eight high origin radial arteries, six were branches of the brachial artery while the remaining two arose from the 3^rd^ part of the axillary trunk. The results are summarized in Table 1.

**Table 1:** Statistical breakdown of sites of origin of the radial artery

Origin	Right	Left	Total
**A continuation of the brachial in the cubital fossa**	29 (46.8%)	25 (40.3%)	54 (87.1%)
**Atypical (high** Brachial artery in arm	4 (6.5%)	2 (3.2%)	6 (9.7%)
**branching) origin from** Axillary artery	1 (1.6%)	1 (1.6%)	2 (3.2%)
**TOTAL**	34 (54.8%)	28 (45.2%)	62

**Figure 2:**
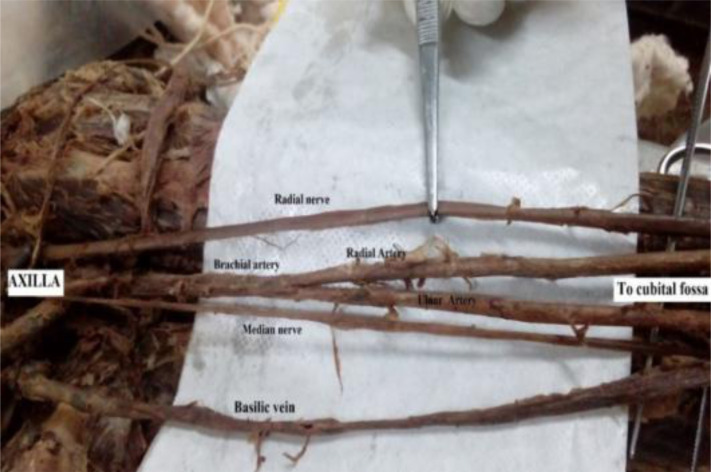
A high origin of the radial artery with a simple brachial division at mid arm level

**Figure 3:**
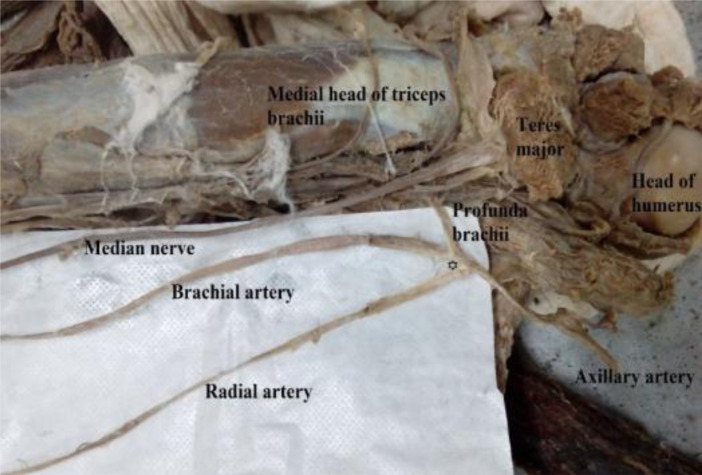
A high origin radial artery arising from the proximal brachial artery at the same level as the profunda brachii

Out of the six radial arteries with their origin in the arm, four (6.5%) were given off by the brachial artery at mid-arm level as a result of a simple high division of the brachial artery (Figure 2). Two of the high origin radial arteries (3.2%) arose from the proximal brachial artery just below the lower border of the teres major. Finally, one (1.6%) of the high origin radial arteries arising from the proximal brachial artery was formed at the same level as the profunda brachii artery (Figure 3). We did not find a radial artery arising from the distal third of the brachial artery.

**Figure 4:**
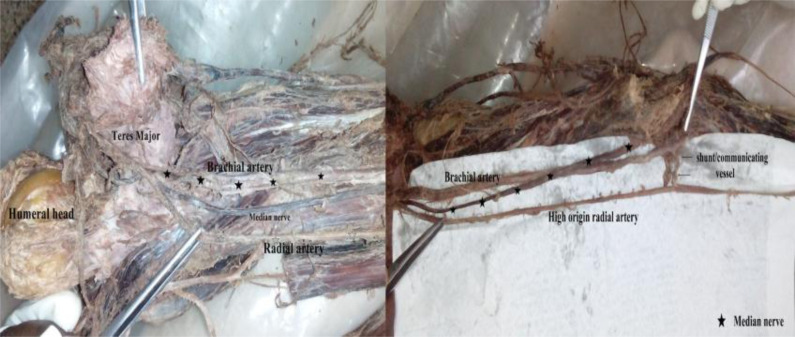
Left side: A high origin radial artery arising from the third part of the axillary artery under cover of the teres major muscle. Right side: showing the radial artery receiving a communicating vessel from the brachial artery within the cubital fossa

**Figure 5:**
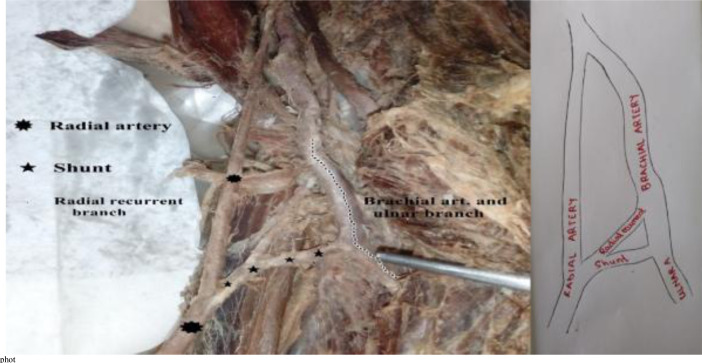
A vascular shunt (anastomosis) from the brachial artery to the radial artery within the cubital fossa. Note the radial recurrent arising from the shunt

In two of the dissections, we observed a radial artery arising from the third part of the axillary artery under cover of the teres major muscle (Figure 4). This atypical radial artery ran next to the brachial continuation within the arm with the median nerve passing between these two as it crossed over the latter to get to its medial position in the cubital fossa. Within the cubital fossa the atypical radial artery received a shunt/communicating vessel from the brachial artery (Figure 4). A radial recurrent branch was given from the shunt and ran into the plane between brachialis and brachioradialis muscles (Figure 5).

## Discussion

Approximately one fifth of any population will show variations in the upper limb arterial system (18). These variations present unique problems during intravenous instrumentation, while also being of great clinical importance in surgeries and the interpretation of radiological images. A majority of the variations in the upper limb arterial system involve the radial artery with a good proportion touching on its origin (4,19). Other variations are relatively uncommon and include duplication, anastomoses, arterial loops and absence of the radial artery (6,7).

In this study, the radial artery arose from the brachial artery within the cubital fossa in 87.1% (54/62) of our study population. This observation is in accord with descriptions in common texts and various authors as the typical origin of the brachial artery (1,2,4). We observed a high origin radial artery in only 12.9% (8/62) of our sample population. This is comparable to reports of a high origin radial artery being present in up to 15% of cadaveric specimens (15–17). Slightly lower prevalences of 5.1% and 8.8% have been reported in angiographic studies (12–14). This disparity remains largely unexplained. It is, however, possible that the lower prevalences in the latter are due to sampling bias, as subjects requiring angiography are a specific sub population that suffers cardiac or vascular related diseases requiring catheterization and therefore do not represent the general population.

Out of the eight high origin radial arteries, six (9.7%) were branches of the brachial artery while the remaining two (3.2%) arose from the 3^rd^ part of the axillary trunk. This finding was consistent with reports that a high origin radial artery commonly arises from the brachial trunk at frequencies of 8.6% and 7.7%, respectively (13,19). In a study carried out on 750 upper limbs, McCormack et al. reported brachioradial artery originating from the brachial artery at frequency of 7.5% (9). In the current study, we observed that two-thirds of the high origin arteries arising from the brachial artery did so from its middle third, in keeping with the findings of Deepa and Martin, 2016 and Shetty, 2012 (17,20). Although based on a review paper that pooled data from 11 papers found that the brachioradial artery originates more frequently from the upper third (71.5%) of the brachial, followed by the middle (19.3%) and inferior thirds (9.2) respectively (10). Observations as to the site of origin of the ‘high origin radial artery’ on the brachial artery are contradictory. Perlin et al, 2006 highlighted the varied reports regarding the commonest site of high origin of the radial artery with some authors citing the proximal brachial artery and others the middle portion of the brachial artery (21). It is possible that the disparity stems from differences in description, where some authors are using the distance of origin of the radial artery from the intercondylar line, the distance of origin of the radial artery from the profunda brachii (21) and the origin of the radial artery from either the proximal, mid or distal portions of the brachial artery (17,20,22,23). It is plausible that distance of origin of the radial artery as measured from the intercondylar line is influenced considerably by the length of the arm and the use of the origin of profunda brachii as an anatomical landmark may be unreliable, since there are reports of variance in the origin of the profunda brachii (17,22). Nonetheless, both the ‘sequential sprouting’ and ‘vascular labyrinth’ theories of arterial development in limbs put forward a possibility of arterial origins being variable (3) and as such it should be anticipated that a high origin radial artery may arise at any length of the brachial artery.

In our study, only two vessels had their origin at the axillary artery constituting 3.2% of our sample. The prevalence of an axillary origin of the radial artery in our study was comparable to these reports (8,9,13,19,21), which reported prevalences of 1%, 2.3%, 1.67%, 1.2%, and 2.1% respectively. The slightly higher prevalence in our study was in accord with a previous observation that an axillary origin of the radial artery is commoner in Africans than in Caucasians, at 5% compared to 2.7% (19). There exists no clear explanation to this observation but the role of genes in vascular rearrangements during development may underlie this phenomenon.

We also observed one example of an anastomotic vessel or shunt arising from the distal end of the brachial artery (near the commencement of the ulnar artery) within the cubital fossa and joining a high origin radial artery. The radial recurrent artery (RRA) arose from this anastomotic vessel. Anastomosis between a high origin radial artery and the brachial or ulnar arteries is uncommon. A literature search, to the best of our knowledge, did not yield reports of a similar shunt but revealed multiple different patterns of anastomosis between a high origin radial artery and other arm/forearm vessels as captured in the descriptions of Gruber in 1870. Wysiadecki et al., 2017 outlined the anastomoses between a high origin radial artery and the vestigial median artery or the anterior interosseous (3). Further, Docimo et al., 2009 and Piagkou et al., 2016 reported a high origin radial artery that rejoins the brachial artery in the cubital fossa (15,24). These anastomoses have been described as representations of primitive developmental relationships between forearm vessels that play a crucial role in the final configuration of the definitive vessel, in this case, the radial artery. The anastomotic vessel in our study may represent the second and definitive origin of the radial artery that then allows the stump of the high origin radial artery proximal to it to regress resulting in both the radial and ulnar arteries arising from the brachial artery in the cubital fossa. After full development of the definitive vessel, it is expected that most of these anastomoses regress. However, the prevailing hemodynamics and oxygen/nutrient demands may favour their persistence.

The development of the radial artery involves two sites; an initial vessel from the brachial artery proximal to site of origin of the typical radial artery followed later by a second origin at from the brachial artery at the level of the cubital fossa adjacent to the origin of the ulnar artery. Normally the stump of the radial artery proximal to the second origin undergoes involution and regresses while the second origin joins the distal remaining portion of the initial primitive radial artery and becomes the definitive vessel. However, genetics, hemodynamics, nutritional demands and smooth muscle-endothelial interactions may cause either the proximal radial stump to persist without the second origin forming or both the proximal stump and the second origin persisting to form a high origin radial artery with an anastomotic vessel as evident in this study. With the development of the radial artery happening quite late during in embryogenesis (3,4), developmental delays, arrests or hitches will often manifest as variations. This explains the high prevalence of variations involving the radial artery.

Recurrent radial artery was not a primary focus of the current study. The anatomy of the RRA artery is so variable. McCormack et al. reported variations related to the origin of the recurrent radial arteries observed branched off the typical radial artery in 81.6% of cases, the posterior radioulnar division in 9.2%, directly from the brachioradial artery in 5%, and the cubital crossover in 4.2% (8). Rodriguez-Niedenfuhr et al. concluded that when a brachioradial artery is present, the RRA most commonly originated from it (65%), while in those cases with variations of the other major trunks, the most common origin was the normal radial artery (25).

It is important to note that high ulnar artery could also be observed. Such atypical vessel may arise directly from axillary or brachial artery and courses superficial to the flexors of the forearm (9,26,27). The incidence of this anatomical variation range between 0.67% and 7% (10,28). The median artery (MA) which is also a common arterial variant that accompanies the median nerve. This artery coexists in the whole arterial pattern of the upper limb with a brachial artery proper that branches into radial and ulnar arteries. The MA originating above the elbow level (from the brachial or the axillary artery) is called brachiomedian artery (BMA) (29).

The findings of this study, as well as similar other studies, emphasize how frequently such anatomical variations occur. With the radial artery being utilized during clinical, surgical and radiological interventions so frequently, an increased understanding and anticipation of such topographic variances benefits patient care and helps avoid preventable iatrogenic injury.
